# Supplementary material for: Diet Rich in Simple Sugars Promotes Pro-Inflammatory Response via Gut Microbiota Alteration and TLR4 Signaling
Source: Cells. 2020 Dec 16;9(12):2701. doi: 10.3390/cells9122701 (PMC7766268; doi:10.3390/cells9122701)
Supplement: Supplementary file 1 [file cells-09-02701-s001.pdf]

Supplementary Tables

Table S1. Main components of the diets.

Crude nutrients and moisture	Diet			
	Standard C1324	Control C1000	High-sugar C1010	High-fiber C1014
Crude Protein [mg/kg]	192.111 (19.2%)	172.650 (17.3%)	170.750 (17.1%)	171.490 (17.1%)
Crude Fat [mg/kg]	40.791 (4.1%)	50.830 (5.1%)	50.450 (5%)	50.598 (5.1%)
Crude Fiber [mg/kg]	60.744 (6.1%)	30.970 (3.1%)	15.170 (1.5%)	200.330 (20%)
Crude Ash [mg/kg]	58.553 (5.9%)	54.846 (5.5%)	41.708 (4.2%)	54.707 (5.5%)
Moisture [mg/kg]	113.426 (11.3%)	82.540 (8.3%)	49.870 (5%)	73.492 (7.3%)
Nitrogenfree extractives [mg/kg]	534,374 (53.4%)	608.164 (60.7%)	672.052 (67.2%)	449.383 (45%)

Table S2. Metabolized energy of the diets.

Metabolized energy	Diet			
	Standard	Control	High-sugar	High-fiber
Protein [kcal/kg]	768 (24 %)	691 (20 %)	683 (18 %)	686 (26 %)
Fat [kcal/kg]	367 (11 %)	457 (13 %)	454 (12 %)	455 (17 %)
Carbohydrates [kcal/kg]	2091 (65 %)	2358 (67 %)	2635 (70 %)	1532 (57 %)

Table S3. Saccharide content.

Crude nutrients saccharide content	Diet		
	Control	High-sugar	High-fiber
Sucrose	11,4 %	46,4 %	11,4 %
Corn starch	53 %	15 %	29,8 %

**Dextrose**	-	7 %	-

Table S4. Fluorochrome-labeled antibodies used for flow cytometry.

Epitope – Fluorochrome	Clone	Manufacturer	Cat#	RRID
**Fixable Viability Dye eFluor 780**	-	Thermo Fisher Scientific	65-0865-14	-
**CD3 – FITC**	145-2C11	BioLegend	100306	AB_312671
**CD4 – Brilliant violet 605**	GK1.5	BioLegend	100451	AB_2564591
**CD8 – Brilliant violet 650**	53-6.7	BioLegend	100741	AB_11124344
**CD25 – APC**	PC61.5	Thermo Fisher Scientific	17-0251-82	AB_469366
**Foxp3 – PE**	FJK-16s	Thermo Fisher Scientific	12-5773-82	AB_465936
**Rorγt – Brilliant violet 421**	Q31-378	BD Biosciences	562894	AB_2687545
**CD3 – eFluor 450**	17A2	Thermo Fisher Scientific	48-0032-82	AB_1272193
**CD11c – Brilliant violet 711**	N418	BioLegend	117349	AB_2563905
**CD45 – Alexa Fluor 700**	30-F11	BioLegend	103128	AB_493715
**CD49b – eFluor 450**	DX5	Thermo Fisher Scientific	48-5971-82	AB_10671541
**CD45R/B220 – Brilliant violet 510**	RA3-6B2	BioLegend	103247	AB_2561394
**Ly6G – FITC**	1A8	BioLegend	127606	AB_1236494
**Ly6C – Brilliant violet 605**	HK1.4	BioLegend	128036	AB_2562353
**iNOS – PE-Cy7**	CXNFT	Thermo Fisher Scientific	25-5920-80	AB_2573499

Table S5. PCR primers used for gene expression analysis by quantitative RT-PCR.

Gene	Forward	Reverse	Reference
Rps12	CCTCGATGACATCCTTGGCCTGAG	GGAAGGCATAGCTGCTGGAGGTGT	[1]
Tgfb1	TTGCTTCAGCTCCACAGAGA	TGGTTGTAGAGGGCAAGGAC	This study
Tnf	TCTCTTCAAGGGACAAGGCT	CGGACTCCGCAAAGTCTAAG	[2]
Il1b	CTTCAGGCAGGCAGTATCAC	TCCATTGAGGTGGAGAGCTT	[2]

Nos2	TCAGCCAAGCCCTCACCTAC	CCAATCTCTGCCTATCCGTCTC	[3]
Irak3	TTGGTCCTGGGCACAGAAA	AATAGCTCGACGATGTCCCAT	[4]
Eef2	CTGACACTCGCAAGGATGAG	CCCGGTCCATCTTGTTCATC	This study
Reg3b	CTGCCTTAGACCGTGCTTTC	CCCTTGTCCATGATGCTCTT	[5]
Reg3g	TTCCTGTCCTCCATGATCAAAA	CATCCACCTCTGTTGGGTTCA	[6]
Il22	CAACTTCCAGCAGCCATACA	GTTGAGCACCTGCTTCATCA	[7]
Clec7a	GCCCTGTGAAGCAATGAAAT	TAGGAAGGCAAGGCTGAGAA	This study
Clec4n	TGAGCCCTTTCTCCTTCTGA	CCACCTGGACCCTCTTACAA	This study
Nfkbiz	CAGAGAGGCCCCTTTCAAGG	TGGCGAACATCTTCCATGCT	This study
Tlr4	GGTGAGAAATGAGCTGGTAA	CGGCTCTGAATAAAGTGTCT	This study
Il17f	TGTTGGGACTTGCCATTCTG	ATTGATGCAGCCTGAGTGTC	This study
Lcn2	TGGAAGAACCAAGGAGCTGT	GGTGGGGACAGAGAAGATGA	[8]
Tjp1	CCACCTCTGTCCAGCTCTTC	CACCGGAGTGATGGTTTTCT	[9]
Ocln	CCTCCACCCCCATCTGACTA	TCGCTTGCCATTCACTTTGC	This study

Supplementary figures

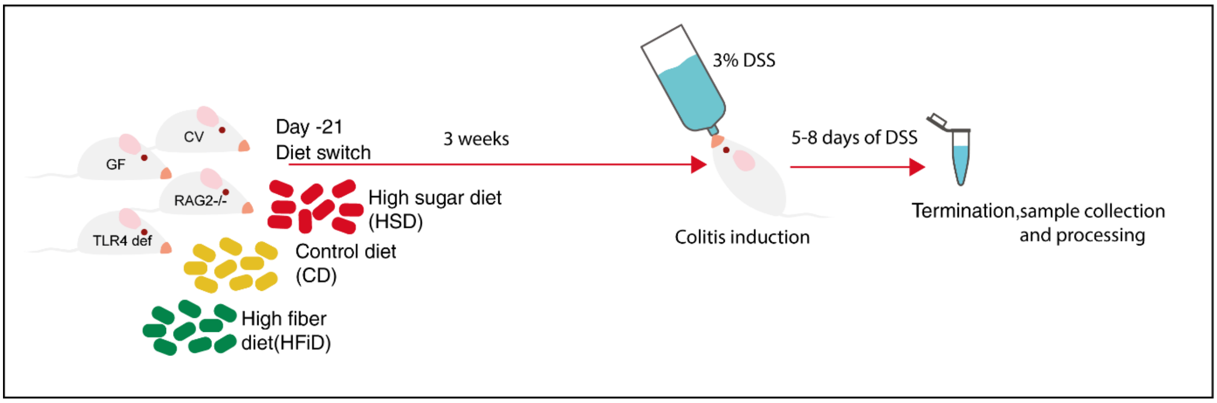

Figure S1. Experimental scheme.

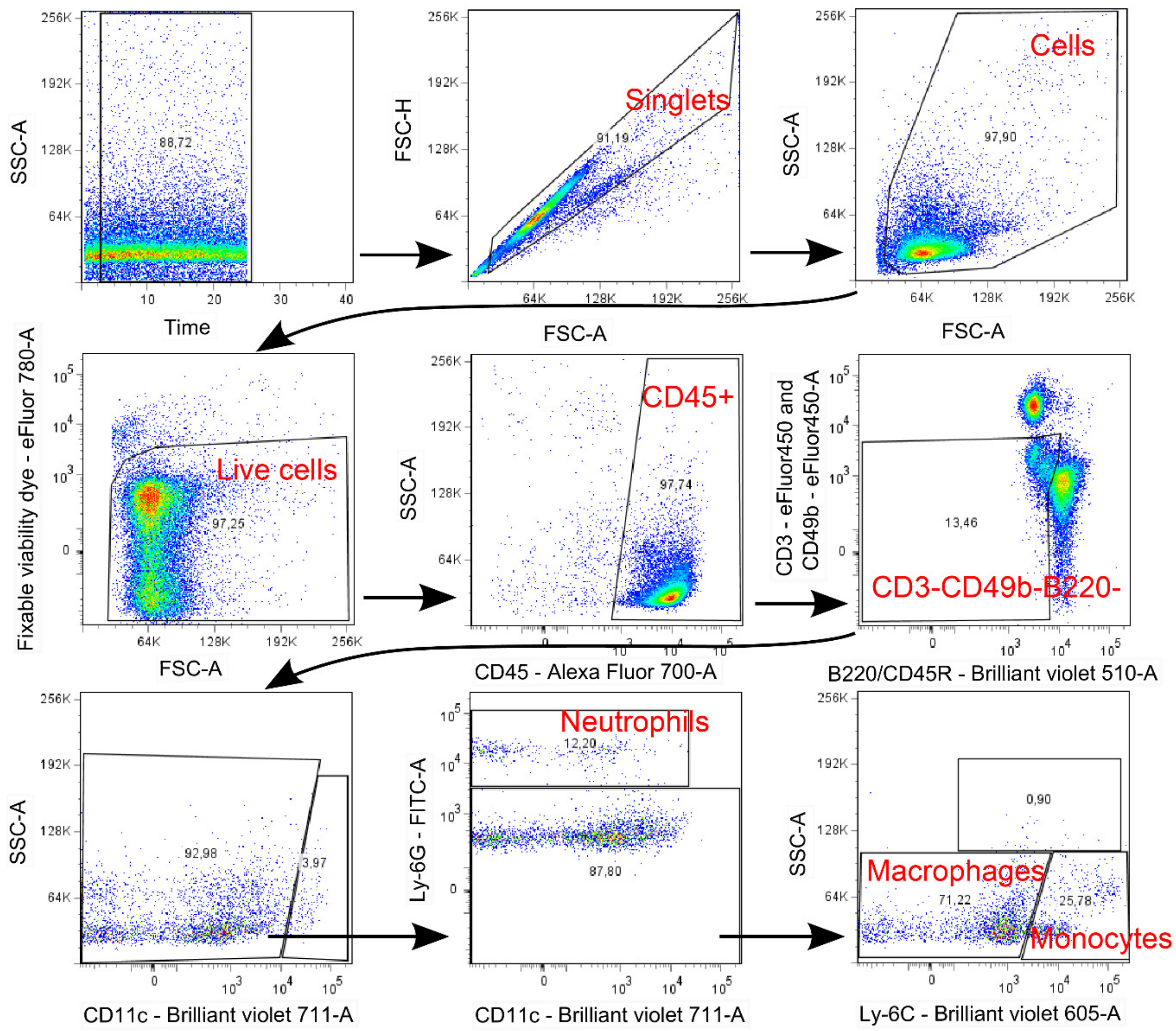

Figure S2. Gating strategy for flow cytometry analysis of innate immune cells.

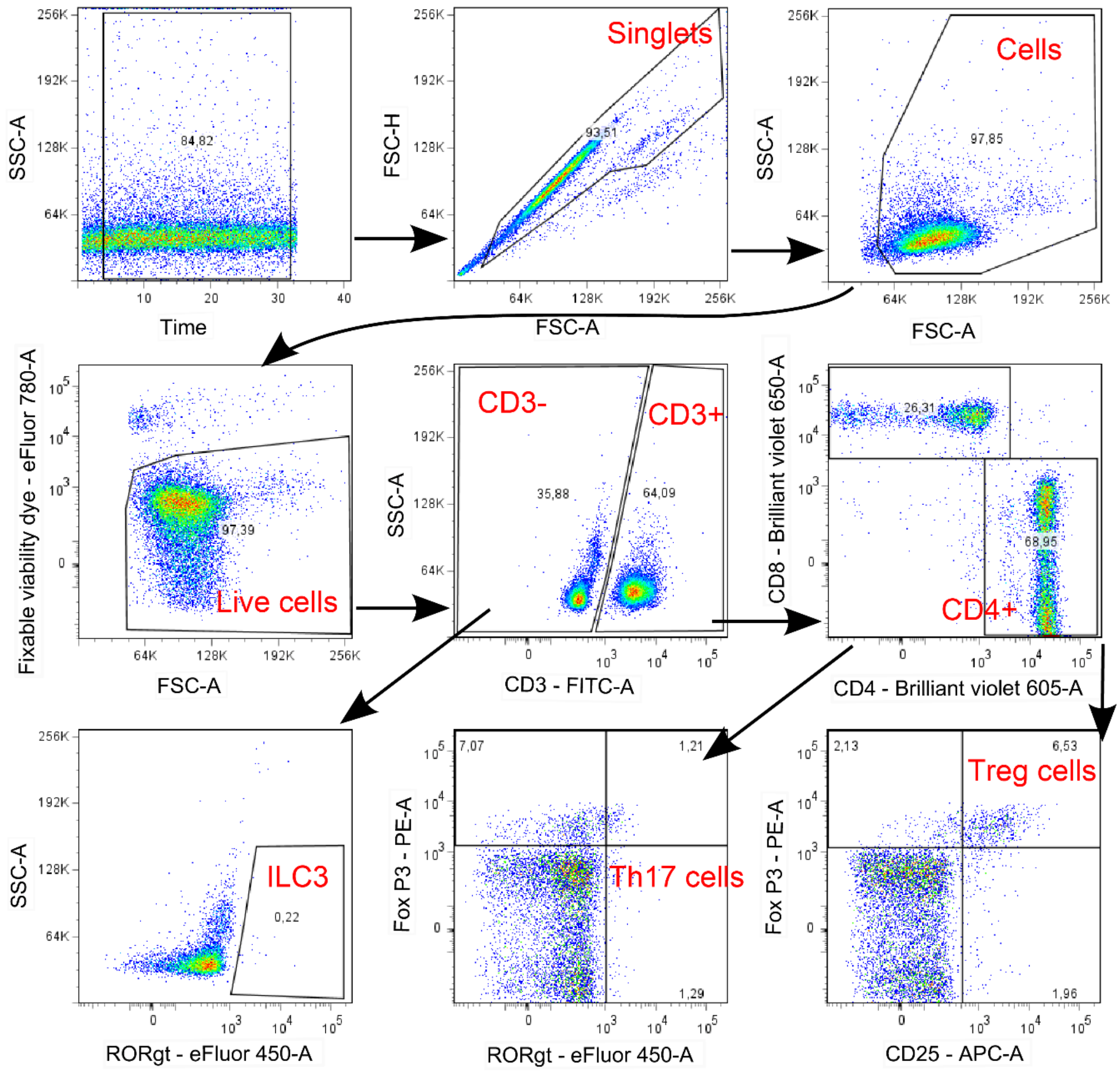

Figure S3. Gating strategy for flow cytometry analysis of T cells and innate lymphoid cells 3 (ILC3).

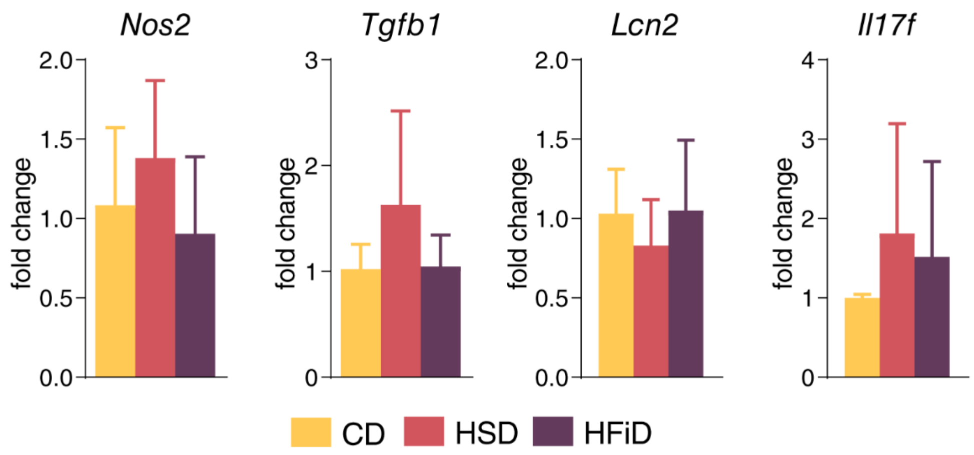

Figure S4. The mRNA expression of *Nos2* (iNos), *Tgfb1*, *Lcn2* (Lipocalin 2) and *Il17f* in colon of healthy mice. Data are presented from one representative experiment out of three, n=5-8 mice per group. Data were analyzed by one-

way ANOVA with Dunnet's multiple comparison test. Data are shown as mean ± SD. CD = control diet, HSD = diet rich in simple carbohydrates, HFiD = diet rich in fiber.

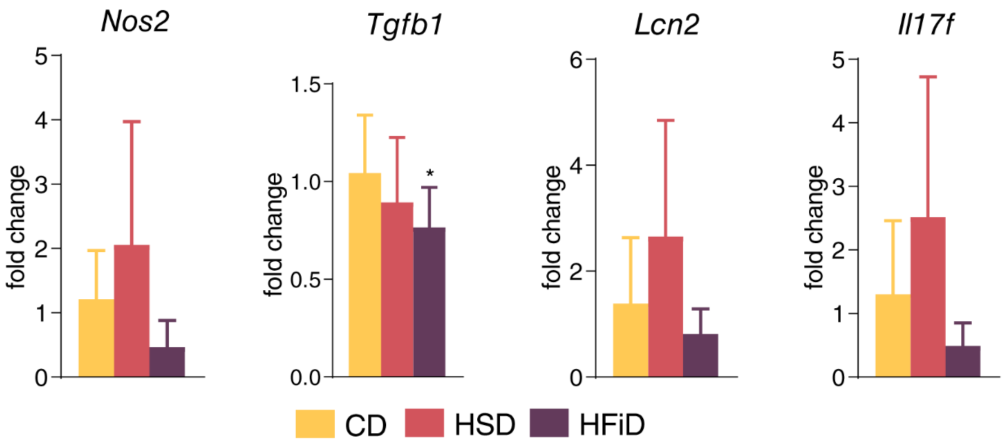

Figure S5. The mRNA expression of *Nos2* (iNos), *Tgfb1*, *Lcn2* (Lipocalin 2) and Il17f in colon of mice suffering acute DSS colitis. Data are presented from one representative experiment out of three, n=5-8 mice per group. Data were analyzed by one-way ANOVA with Dunnet's multiple comparison test. Data are shown as mean ± SD. *p<0.05. CD = control diet, HSD = diet rich in simple carbohydrates, HFiD = diet rich in fiber.

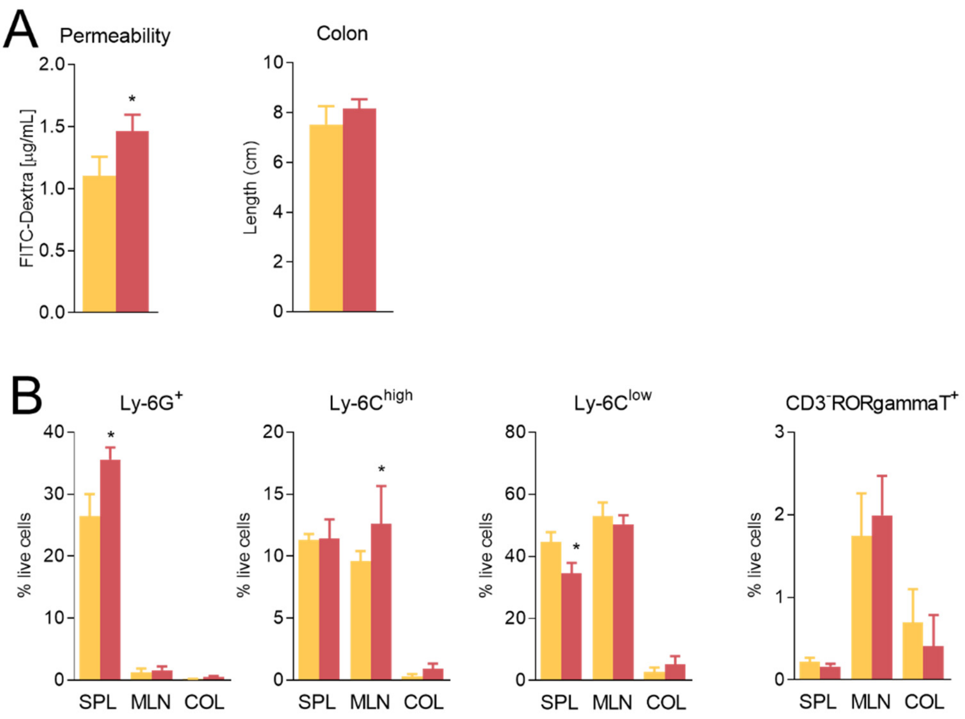

Figure S6. Simple sugars significantly increase intestinal permeability and promote local and systemic pro-inflammatory tuning in healthy RAG2 -/- mice. A) Intestinal permeability and colon length. B) Percentage of live cells from spleens, mesenteric lymph nodes and colons measured by flow cytometry. Data presented are from two experiments, n=5-9 mice per group. Data were analyzed by unpaired t test in respective organ. Data are shown as mean ± SD. *p<0.05. CD = control diet, HSD = diet rich in simple carbohydrates.

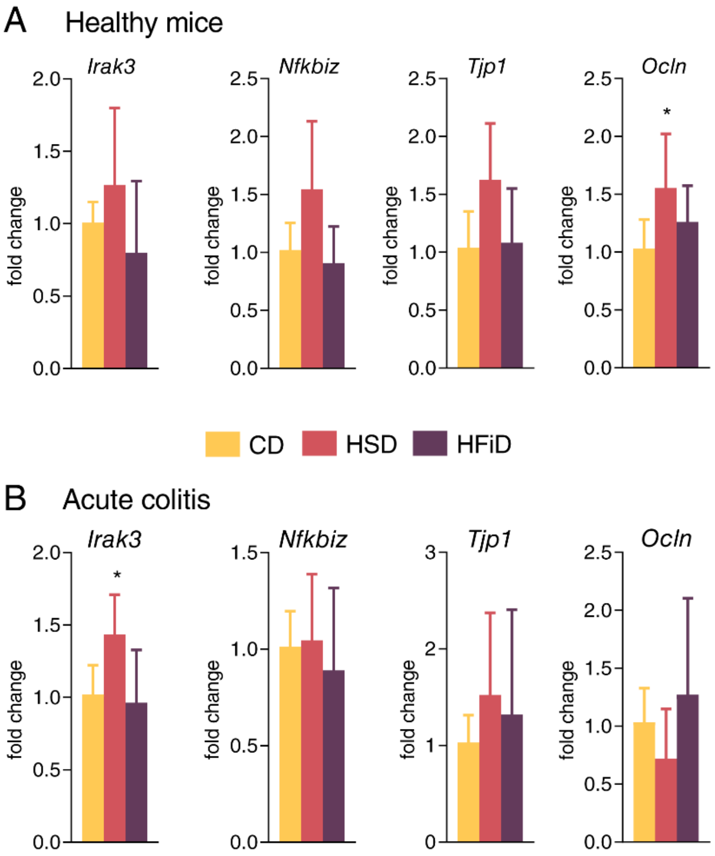

Figure S7. The mRNA expression of pattern recognition receptors Dectin 2 (*Clec4n*) and Toll-like receptor 4 (*Tlr4*), NF-κB pathway regulators IRAK-3 (*Irak3*) and NF-kappa-B inhibitor zeta (*Nfkbiz*), and gut barrier proteins ZO-1 (*Tjp1*) and Occludin (*Ocln*). A) HSD significantly influences the mRNA expression of Occludin (*Ocln*) in colon of healthy mice. B) DSS-induced acute colitis led to significantly higher expression of *Irak3* and only partially influenced the mRNA expression of other genes with enhancement of *Tlr4* and reduction of *Nfkbiz* and *Ocln*. Data were analyzed by one-way ANOVA with Dunnet's multiple comparison test. Data are presented from one representative experiment out of three, n=5-8 mice per group. Data are shown as mean ± SD. *p<0.05. CD = control diet, HSD = diet rich in simple carbohydrates, HFiD = diet rich in fiber.

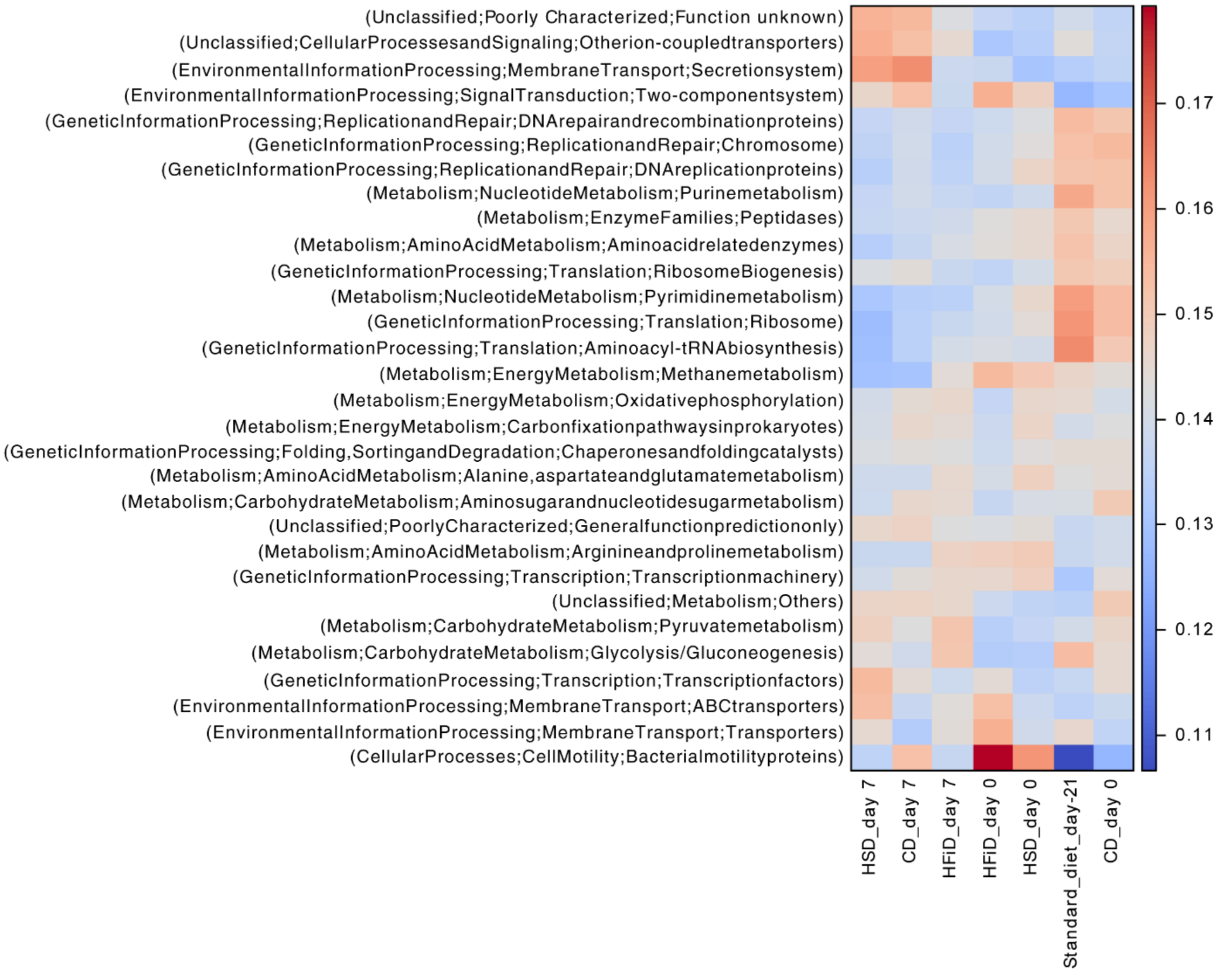

Figure S8. Prediction of metabolical pathways. PICRUSt analysis was used to associate microbiota abundances with metabolic pathways in mice feces on day -21 (before diet switch), day 0 (before DSS treatment) and day 7 of DSS colitis. CD = control diet, HSD = diet rich in simple carbohydrates, HFiD = diet rich in fiber.

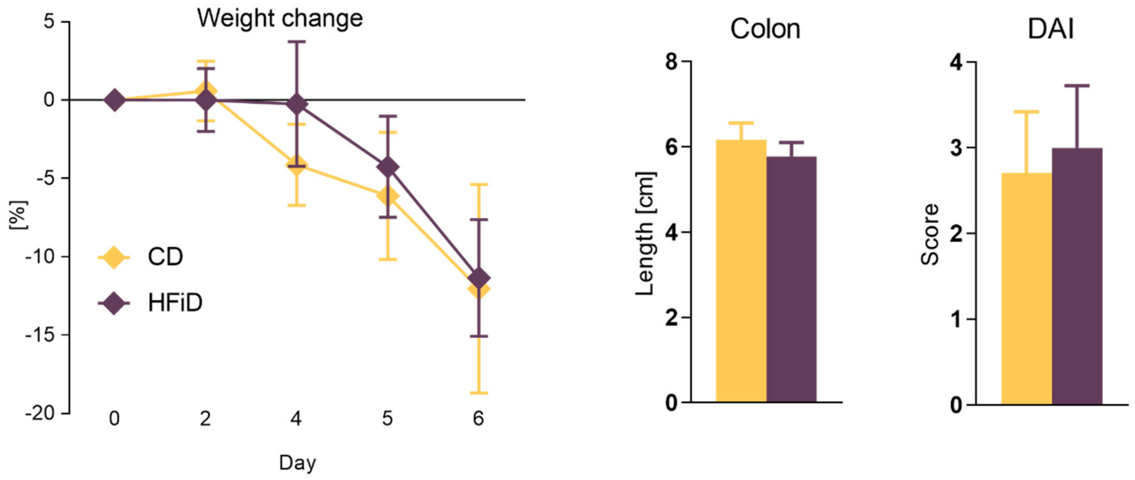

Figure S9. Protective effect of HFiD in acute colitis is not transferred by microbiota alone. Weight change, colon length and disease activity index (DAI) are shown. Data presented are from two experiments, n=5-8 mice per

group. The weight loss was analyzed by two-way ANOVA with Bonferroni post-test and the other parameters by unpaired t test. Data shown as mean ± SD. CD = control diet, HFiD = diet rich in fiber.

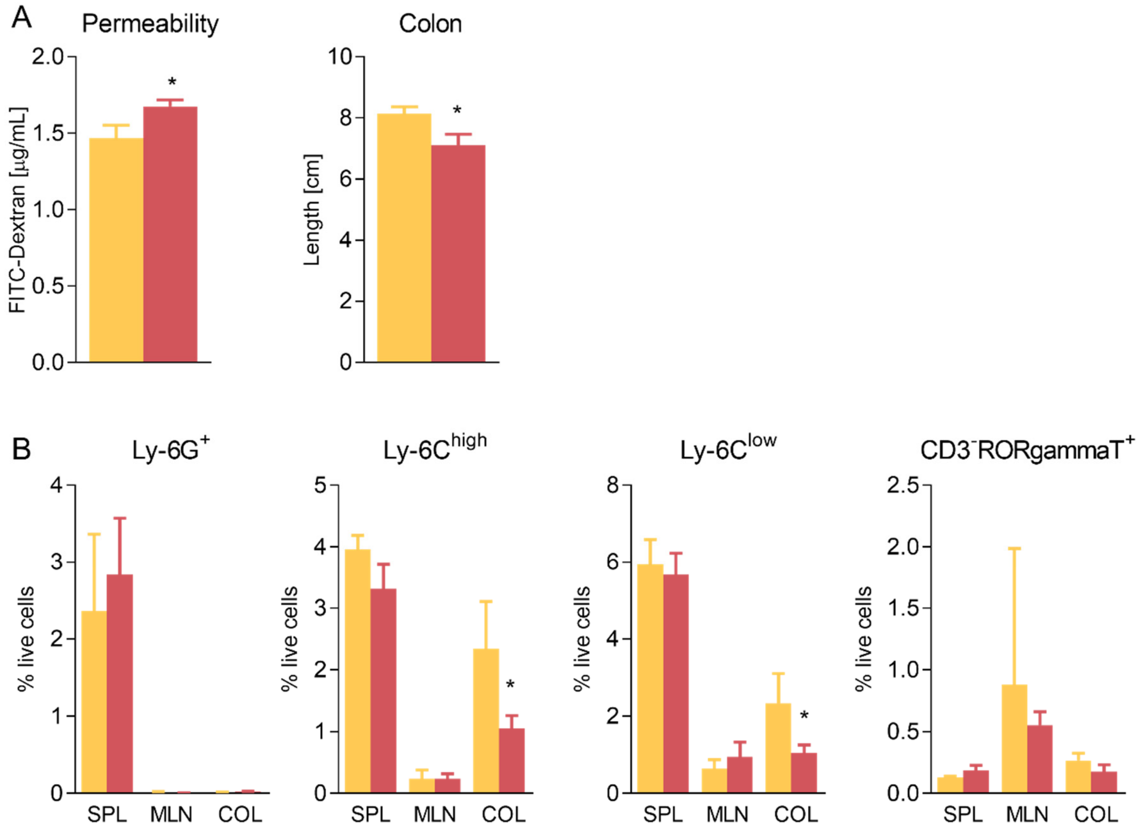

Figure S10. Healthy TLR4 deficient mice. A) Intestinal permeability and colon length. B) Percentage of live cells from spleens, mesenteric lymph nodes and colons measured by flow cytometry. Data presented are from one experiment. Data were analyzed by unpaired t test in respective organ. Data are shown as mean ± SD. *p<0.05. CD = control diet, HSD = diet rich in simple carbohydrates.

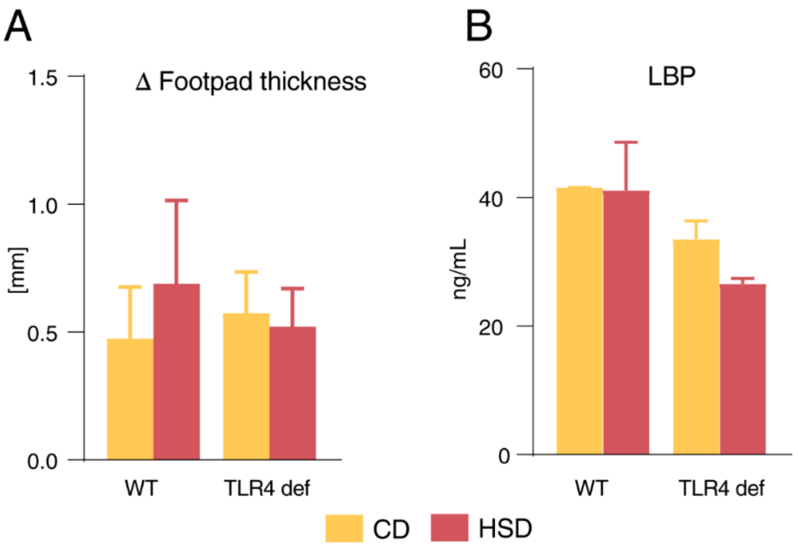

Figure S11. Inflammatory response in TLR4 deficient mice. A) Increase in footpad thickness 24 hours after λ-carrageenan intradermal injection in wild-type and TLR4 deficient mice. B) LPS binding protein levels in mouse serum in WT and TLR4 deficient mice. Data presented are from two experiments, n=5-8 mice per group. Data were analyzed by unpaired t test. Data are shown as mean ± SD. CD = control diet, HSD = diet rich in simple carbohydrates, LBP = LPS binding protein.

Supplementary references

1. Klimesova, K.; Kverka, M.; Zakostelska, Z.; Hudcovic, T.; Hrncir, T.; Stepankova, R.; Rossmann, P.; Ridl, J.; Kostovcik, M.; Mrazek, J.; et al. Altered gut microbiota promotes colitis-associated cancer in IL-1 receptor-associated kinase m-deficient mice. *Inflamm. Bowel Dis.* **2013**, *19*, 1266–1277, doi:10.1097/MIB.0b013e318281330a.

2. Kostovcikova, K.; Coufal, S.; Galanova, N.; Fajstova, A.; Hudcovic, T.; Kostovcik, M.; Prochazkova, P.; Zakostelska, Z.J.; Cermakova, M.; Sediva, B.; et al. Diet rich in animal protein promotes pro-inflammatory macrophage response and exacerbates colitis in mice. *Front. Immunol.* **2019**, *10*, doi:10.3389/fimmu.2019.00919.

3. Tajima, T.; Murata, T.; Aritake, K.; Urade, Y.; Michishita, M.; Matsuoka, T.; Narumiya, S.; Ozaki, H.; Hori, M. EP 2 and EP 4 receptors on muscularis resident macrophages mediate LPS-induced intestinal dysmotility via iNOS upregulation through cAMP/ERK signals. *Am. J. Physiol. - Gastrointest. Liver Physiol.* **2012**, *302*, G524, doi:10.1152/ajpgi.00264.2011.

4. Standiford, T.J.; Kuick, R.; Bhan, U.; Chen, J.; Newstead, M.; Keshamouni, V.G. TGF-B-induced IRAK-M expression in tumor-associated macrophages regulates lung tumor growth. *Oncogene* **2011**, *30*, 2475–2484, doi:10.1038/onc.2010.619.

5. Jacobson, A.; Lam, L.; Rajendram, M.; Tamburini, F.; Honeycutt, J.; Pham, T.; Van Treuren, W.; Pruss, K.; Stabler, S.R.; Lugo, K.; et al. A Gut Commensal-Produced Metabolite Mediates Colonization Resistance to Salmonella Infection. *Cell Host Microbe* **2018**, *24*, 296-307.e7, doi:10.1016/j.chom.2018.07.002.

6. Syder, A.J.; Oh, J.D.; Guruge, J.L.; O'Donnell, D.; Karlsson, M.; Mills, J.C.; Björkholm, B.M.; Gordon, J.I. The impact of parietal cells on Helicobacter pylori tropism and host pathology: An analysis using gnotobiotic normal and transgenic mice. *Proc. Natl. Acad. Sci. U. S. A.* **2003**, *100*, 3467–3472, doi:10.1073/pnas.0230380100.

7. Molinero, L.L.; Cubre, A.; Mora-Solano, C.; Wang, Y.; Alegre, M.L. T cell receptor/CARMA1/NF-κB signaling controls T-helper (Th) 17 differentiation. *Proc. Natl. Acad. Sci. U. S. A.* **2012**, *109*, 18529–18534, doi:10.1073/pnas.1204557109.

8. Stienstra, R.; Mandard, S.; Patsouris, D.; Maass, C.; Kersten, S.; Müller, M. Peroxisome Proliferator-Activated Receptor α Protects against Obesity-Induced Hepatic Inflammation. *Endocrinology* **2007**, *148*, 2753–2763, doi:10.1210/en.2007-0014.

9. Volynets, V.; Rings, A.; Bárdos, G.; Ostaff, M.J.; Wehkamp, J.; Bischoff, S.C. Intestinal barrier analysis by assessment of mucins, tight junctions, and α-defensins in healthy C57BL/6J and BALB/cJ mice. *Tissue Barriers* **2016**, *4*, doi:10.1080/21688370.2016.1208468.
